# Ischemia monitoring in off-pump coronary artery bypass surgery using intravascular near-infrared spectroscopy

**DOI:** 10.1186/1749-8090-1-12

**Published:** 2006-05-24

**Authors:** Franziska H Bernet, David Reineke, Hans-Reinhard Zerkowski, Doan Baykut

**Affiliations:** 1 Division of Cardio-Thoracic Surgery, University Hospital Basel, Switzerland

## Abstract

**Background:**

In off-pump coronary artery bypass surgery, manipulations on the beating heart can lead to transient interruptions of myocardial oxygen supply, which can generate an accumulation of oxygen-dependent metabolites in coronary venous blood. The objective of this study was to evaluate the reliability of intravascular near-infrared spectroscopy as a monitoring method to detect possible ischemic events in off-pump coronary artery bypass procedures.

**Methods:**

In 15 elective patients undergoing off-pump myocardial revascularization, intravascular near-infrared spectroscopic analysis of coronary venous blood was performed. NIR signals were transferred through a fiberoptic catheter for signal emission and collection. For data analysis and processing, a miniature spectrophotometer with multivariate statistical package was used. Signal acquisition and analysis were performed before and after revascularization. Spectroscopic data were compared with hemodynamic parameters, electrocardiogram, transesophageal echocardiography and laboratory findings.

**Results:**

A conversion to extracorporeal circulation was not necessary. The mean number of grafts per patient was 3.1 ± 0.6. An intraoperative myocardial ischemia was not evident, as indicated by electrocardiogram and transesophageal echocardiography. Continuous spectroscopic analysis showed reproducible absorption spectra of coronary sinus blood. Due to uneventful intraoperative courses, clear ischemia-related changes could be detected in none of the patients.

**Conclusion:**

Our initial results show that intravascular near-infrared spectroscopy can reliably be used for an online intraoperative ischemia monitoring in off-pump coronary artery bypass surgery. However, the method has to be further evaluated and standardized to determine the role of spectroscopy in off-pump coronary artery bypass surgery.

## Background

The intraoperative management of hemodynamic changes in off-pump coronary artery bypass surgery (OPCAB) is still a challenge [1-3]. Altered coronary perfusion by manipulation on the beating heart can lead to an interruption of myocardial oxygen supply in the affected areas with metabolic changes and electrophysiologic disturbances followed by a reduction of contractility [4]. Conventional intraoperative monitoring methods such as electrocardiogram (ECG), transesophageal echocardiography (TEE) or pulmonary arterial pressure are routinely used to gain information about acute myocardial ischemia. However, luxation or repositioning of the heart for assembly of coronary anastomoses can easily lead to a transient loss of signal, interrupting the continuous monitoring with these instruments [5].

Near-infrared (NIR) spectroscopy is an appropriate method for ischemia monitoring in myocardial tissue [6-9]. An acute deterioration of coronary blood supply can result in a release of ischemic metabolites, predominantly oxygen-dependent chromophores into the coronary sinus. These metabolites are detectable in the coronary sinus blood, often prior to the appearance of substances representing cellular injury such as troponins and creatine kinase (CK-MB) in the peripheral blood. The online intravascular NIR spectroscopic analysis of the coronary sinus blood with a fiberoptic catheter allows a direct ischemia monitoring at myocardial level [10].

The aim of our study was to evaluate the clinical reliability of myocardial ischemia monitoring by using intravascular NIR spectroscopy in OPCAB surgery. The study protocol was approved and accepted by the ethics committee of the University of Basel and written/informed consent was obtained.

## Methods

### Patients

Fifteen patients, 13 male and 2 female, with a mean age of 61.3 ± 12.2 years, underwent elective OPCAB surgery. Twelve patients (80%) had a three vessel disease and 3 patients (20%) had a two vessel disease with a proximally occluded left anterior descending coronary artery. Patients with preoperative poor left ventricular function, rhythm disturbances and anticipated concomitant interventions were excluded from the study. There were no redo procedures. Preoperative patient characteristics are shown in Table 1.

**Table 1:** Preoperative Patient Characteristics

Variable	N = 15
Gender: male (%)	80 (12)
Age (yrs)*	61.3 ± 12.2
BMI (Kg/m^2^)*	26.8 ± 4.6
3 VD (%)	80 (12)
	
Art. Hypertension (%)	60 (9)
NIDDM/IDDM (%)	33 (5)
Dyslipidemia (%)	73 (11)
Smoking history (%)	60 (9)
COPD (%)	7 (1)
Family history (%)	47 (7)
	
Preoperative MI (<3 mts) (%)	40 (6)
Ejection fraction [%]*	62.3 ± 7.8
Euroscore*	3.0 ± 3.4

*Values are mean ± SD

Values in brackets are the number of patients

### NIR spectroscopy

Near-infrared (NIR) is defined as the wavelength interval between 700 nm and 2000 nm. The good penetration of the NIR light into soft tissue makes the NIR spectroscopy appropriate for in-vivo detection of blood and/or tissue-bound oxygen-dependent chromophores such as hemoglobin, myoglobin and cytochromes [6,11,12]. For spectroscopic analysis of tissue under in-vivo-conditions, specific light diodes (optodes) are required which usually work in a dual configuration with emitting and collecting light transmitted through the tissue sample [13]. Collected NIR light is transferred by a fiberoptic cable to a photomultiplier and converted into an electric signal.

**Figure 1:**
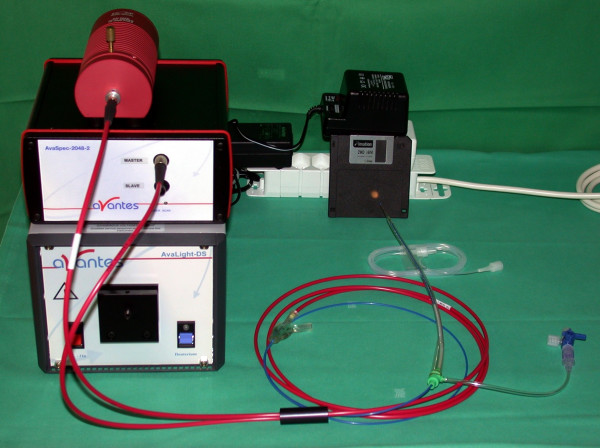
NIR spectroscopy: The fiberoptic catheter is connected to the spectrophotometer and the halogen light source (red).

Catheter-linked NIR spectroscopic analysis is a novel technique in which emitting and collecting optodes are positioned at the same location, allowing that NIR signals can be transferred to virtually any intravascular region [10]. Both emitting and collecting fibers of the catheter are located in a concentric array. The electronic conversion of the difference between emitted and collected NIR light provides the actual absorption spectra of oxygenated and de-oxygenated chromophores. For illumination, a halogen light source is used. The acquisition and analysis of NIR signals were performed with an AvaSpect-2048 spectrometer (Avantes BV, Eerbeek, Netherlands) (Figure 1).

**Figure 2:**
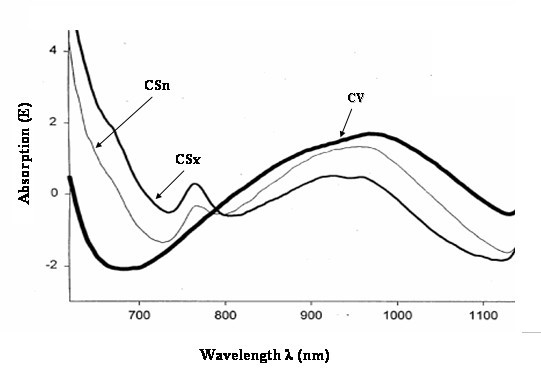
The intravascular application of NIR spectroscopy was evaluated in acute myocardial ischemia-reperfusion experiments in animals using the coronary sinus access. Specific NIR absorbance changes during myocardial ischemia. CV, central venous sample. CSn, normal control sample from the coronary sinus blood. CSx, ischemia-related change of NIR spectra pattern after occlusion of the left anterior descending artery. (from: Baykut et al. 2001^10^)

The spectrometric identification of ischemic and non-ischemic tissues was based on the algorithm as described in the literature [14]. For reference in absorbance changes during ischemia, results from in-vitro and animal experiments in acute ischemic settings were used [10] (Figure 2). Measurements were carried out before surgery (baseline), after completing distal anastomoses, and after complete revascularization with all central anastomoses assembled. The collection time for NIR signals from the coronary sinus was 5 minutes at each step of the revascularization.

### Intraoperative management

Anesthesia was induced with Thiopental 5 mg/kg, Pancuronium 0.1 – 0.15 mg/Kg and Fentanyl 1.5 – 5.0 μg/Kg. For maintenance, inhalative anesthesia with Isoflurane 1 Vol% and ventilation with air and 50 Vol% oxygen were used. During assembly of distal anastomoses, oxygen was elevated to 80 Vol%. 300 U/kg of heparin was administered for anticoagulation with a targeted activated clotting time of 400 seconds. For hemodynamic stability, volume substitution with infusions and low dose inotropic support were carried out. The use of pre-warmed intravenous fluids and a heated matress with a warm air blanket helped maintain normothermia.

For systemic pressure monitoring, left radial artery and for pulmonary arterial pressure monitoring, a Swan-Ganz catheter were used. To detect wall motion abnormalities, TEE was performed in all patients at the beginning and the end of the procedure.

### Operative technique

All procedures were performed via median sternotomy. Pedicled left internal thoracic artery (LITA) and saphenous veins were used as bypass grafts. For stabilization of the heart and visualization of coronary arteries, a Guidant Acrobat™ stabilizer (Guidant Corporation, Indianapolis, USA) was used. In all patients, intracoronary shunts were placed. After pericardiotomy, the NIR catheter was inserted into the coronary sinus through the right atrial appendage. For a stable positioning of the catheter in the coronary sinus, a 16 F thin-flex single-stage pediatric venous cannula (Edwards Lifescience, Irvine, CA, USA) was used as a sheath. The catheter tip was located in a fixed position at the sheath's end to avoid dislocation of the catheter from the coronary venous backflow and transillumination through the surrounding tissue. Blood samples were collected from the coronary sinus. After acquisition of baseline NIR spectra, the distal anastomoses were assembled, starting with the LITA to the left anterior descending artery (LAD). Simultaneously, blood gas analyses were carried out and the coronary venous lactate concentration was determined. Before and after revascularization, troponin I (cTnI) CK-MB and myoglobin values from the coronary venous blood were measured.

## Results

Operations could be completed as OPCAB procedures without conversion to conventional coronary bypass surgery. The mean operation time was 185.8 ± 23.5 min. Each patient received LITA as an arterial graft to the LAD. The mean number of grafts per patient was 3.1 ± 0.6. Intraoperative complications or hemodynamic instability were not observed. Significant variations of hemoglobin and hematocrit values that could influence spectroscopic measurements were not observed. In the gas analyses from the coronary venous blood, a significant change of the pO_2 _and SO_2_values between baseline and the end of complete revascularization was detected, while other parameters including pH, pCO_2 _and base excess did not significantly differ from the baseline (Table 2). The increase in lactate concentration of coronary sinus blood before and after revascularization was also not significant. Further ischemic parameters including cTnI, myoglobin and CK-MB were significantly elevated at the final measuring point (Table 3).

**Table 2:** Blood Gas Analyses From the Coronary Sinus

Variable	Start Operation N = 15	End Operation N = 15	p-value
Hemoglobin [g/dL]	10.8 ± 1.2	9.8 ± 0.9	0.2
Hematocrit [%]	32.5 ± 3.8	30.2 ± 2.8	0.3
sO2 [%]	45.9 ± 3.8	51.3 ± 4.8	0.01
pH	7.37 ± 0.04	7.35 ± 0.05	0.4
pO2 [mmHg]	26.3 ± 4.1	31.9 ± 5.7	0.01
pCO2 [mmHg]	47.3 ± 4.9	49.2 ± 2.5	0.4
BE [mmol/L]	0.1 ± 1.9	(-)0.4 ± 1.7	0.6

* data is presented as mean ± SD; for comparison the student-t test was used

**Table 3:** Ischemic Parameters From the Coronary Sinus Blood

Variable*	Start Operation	End Operation	p-value
Troponin I [<2.0 μg/L]	0.3 ± 0.2	2.2 ± 0.7	p < 0.001
CK-MB [5.0 μg/L]	0.7 ± 0.4	2.1 ± 1.3	p = 0.003
Myoglobin [<93 μg/L]	79 ± 20.8	171 ± 45.2	p = 0.001
Lactate [<1.8 mmol/L]	1.3 ± 0.8	1.5 ± 0.5	p = 0.6

* data are presented as mean ± SD; for comparison the student-t test was used *CK-MB*, creatine kinase-MB.

**Figure 3:**
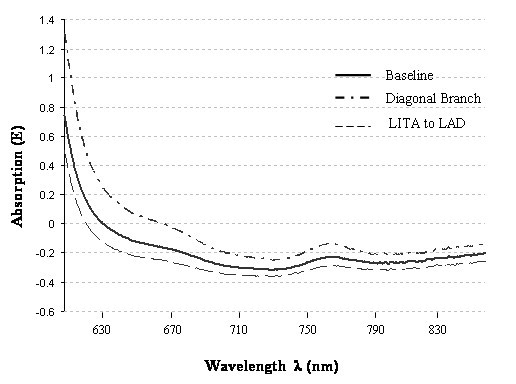
NIR spectra of a patient receiving a distal venous anastomosis to a diagonal branch prior to the LITA – LAD anastomosis. At 760 nm a slight increase (deoxy-hemoglobin) was registered. Decrease of the curve after completing the arterial anastomosis to the LAD with the LITA graft.

Due to uneventful intraoperative courses, changes in NIR absorption spectra observed during the entire surgical procedure were not distinctive enough to identify ischemic myocardial events (Figure 3). In none of the patients, indicators for myocardial ischemia or infarction were observed by using conventional detecting methods.

## Discussion

The usefulness of NIR spectroscopy to reveal in myocardial ischemic episodes was evaluated in several previous studies [7-9]. Parsons et al. used NIR spectroscopy for myocardial ischemia detection in open chest studies with dogs, concluding that the application of NIR spectroscopy is suitable for assessment of the tissue oxygenation relation to contractile function during ischemia [7]. Similarly, Thorniley et al. and Kupriyanov et al. examined changes in hemoglobin oxygenation to study tissue perfusion and flow in pigs with NIR spectroscopy. They found that NIR spectroscopic imaging of myocardial oxygenation and distribution of an intravascular tracer correlated well with coronary flow, allowing an intraoperative optical assessment of the severity of regional ischemia [8,9]. In the open chest study with dogs of Baykut et al. [10], NIR spectroscopic detection was the first time applied at intravascular level by using a fiberoptic catheter. Although different data acquisition and processing techniques were used in the aforementioned studies, the results demonstrate a broad similarity, confirming the feasibility of the NIR spectroscopy for online monitoring of myocardial ischemic events.

Our study was aiming to apply the intravascular NIR spectroscopy at clinical level for the first time. Particularly in OPCAB procedures, transient ischemic episodes of working myocardium can usually be expected. Since the uneventful intraoperative course in all of our patients caused only minor changes of NIR spectra, a conclusive analysis becomes difficult. Remarkable differences were registered only in two patients who initially received a saphenous vein graft anastomosis to a diagonal branch prior to bypassing LAD with LITA. In our opinion, this effect was originating from the position of the epicardial stabilizer in order to anastomose the diagonal branch, resulting in a local perfusion disturbance in the LAD prior to bypassing this vessel. This observation is in accordance with the study of Wang et al., evaluating TEE for ischemia monitoring by detecting new segmental wall abnormalities. He found that the placement of an epicardial stabilizer alone can induce abnormalities in both TEE and ECG indicating an ischemic development [5].

It is difficult to say that the changes in NIR spectra are generated by differences in the oxygenation status of myocardial tissue since the pO_2 _levels in the coronary sinus blood increased, showing no appropriate relationship with NIR absorbance. In contrast, ischemic parameters such as cTnI, CK-MB and myoglobin were elevated at the end of the revascularization, making an at least transient injury at the cellular level evident. However, this increase has to be interpreted with caution since our patient group was small. Despite the measurable increase of ischemic markers, clinical parameters indicating any adverse events could not be observed intra- and postoperatively. Consequently, in an uneventful intraoperative course, minor ischemic changes may remain undetected and hard to interprete using NIR spectroscopy. Since the coronary venous blood indicates the global changes of the myocardium, this technique is not yet capable of measuring regional variations of ischemia such as that caused by local occlusion or reperfusion of any particular vessel. In our point of view, more remarkable results could be registered in patients at the state of an aggravating angina pectoris, before hemodynamical changes arise.

### Study limitation

Since the aim of our study was to evaluate the technical feasibility of the intravascular NIR spectroscopy in detecting myocardial ischemia, we deliberately selected low risk patients undergoing OPCAB surgery. We are aware that the basic information we could draw from this pilot clinical study has to be revised before transferred to routine application. Furthermore, for the placement of the catheter into the coronary sinus, an invasive approach is unevitable. Therefore, the intravascular NIR spectroscopy is a monitoring method which should be limited to patients at high risk, such as patients with an acute coronary insufficiency or imminent myocardial infarction.

## Conclusion

Our study with fifteen patients using OPCAB technique indicated that intravascular NIR spectroscopy is technically feasible. Nevertheless, a refinement and standardization of NIR-data acquisition is necessary and more work is required to understand the behaviour of NIR light in myocardial tissue if NIR spectroscopy is to be used as a reliable clinical monitoring method.

## Abbreviations

OPCAB: off-pump coronary bypass

ECG: electrocardiogram

TEE: transesophageal echocardiography

NIR: near-infrared

CK-MB: creatine kinase

LAD: left anterior descending

LITA: left internal thoracic artery

cTnI: cardiac troponin I

## Competing interests

The author(s) declare that they have no competing interests.

## Authors' contributions

The authors declare that they have made substantive intellectual contributions to the conception, design and data acquisition of the study. All authors have been involved in preparing and revising the manuscript and have given the final approval before submitting the manuscript.
